# 

**Published:** 1870-10

**Authors:** 


☞ Send for a Copy of W. B. KEEN & COOKE'S New Classified Price List of Medical Books. Just published. Mailed free.

